# Detection of MicroRNA in Hepatic Cirrhosis and Hepatocellular Carcinoma in Hepatitis C Genotype-4 in Egyptian Patients

**DOI:** 10.1155/2017/1806069

**Published:** 2017-05-23

**Authors:** Hala M. Demerdash, Hend M. Hussien, Ehab Hassouna, Emad A. Arida

**Affiliations:** ^1^Department of Clinical Pathology, Alexandria University Hospitals, Alexandria, Egypt; ^2^Pharmacology and Toxicology Department, Faculty of Pharmacy and Drug Manufacturing, Pharos University, Alexandria, Egypt; ^3^Department of Internal Medicine, Hepatology Unit, Faculty of Medicine, Alexandria University, Alexandria, Egypt; ^4^Department of Anesthesia and Surgical Intensive Care, Alexandria University Faculty of Medicine, Chamblion Street, Elazareeta, District-Zip Code 21526, Alexandria, Egypt

## Abstract

**Background:**

In Egypt, the prevalence of chronic hepatitis C (CHC) infection is 13.8% of whole population and about 80% of the patients with hepatocellular carcinoma have underling hepatitis C.

**Aim:**

This study was designed to assess the diagnostic value of plasma miR-122 and miR-21 in patients with CHC, genotype-4, to detect fibrosis progression versus noninvasive indices and their diagnostic value in detection of early stages of hepatocellular carcinoma (HCC).

**Methodology:**

A prospective study that included 180 patients, divided into 3 groups: healthy controls (group I), CHC patients (group II), and hepatitis C patients with HCC (group III); all cases were subjected to thorough clinical, radiological, and laboratory investigations. Selected biomarkers were evaluated and correlated with degree of liver damage. Results revealed that miR-122 followed by miR-21 had the highest efficiency in prediction of liver cell damage. Also, miR-21 was strongly correlated with vascular endothelial growth factor (VEGF) and alpha fetoprotein (*α*-FP) in HCC patients.

**Conclusions:**

Plasma miR-122 and miR-21 had strong correlation with degree fibrosis in HCV genotype-4 patients; consequently they can be considered as potential biomarker for early detection of hepatic fibrosis. Moreover, miR-21 can be used as a potential biomarker, for early detection of HCC combined with VEGF and *α*-FP.

## 1. Introduction

Chronic infection with hepatitis C virus (HCV) accounts for 170–200 million patients worldwide. It has six major genotypes (types 1–6); genotype-4 represents 12%–15% of total global HCV infection. However, about 90% of Egyptian patients suffering from HCV belong to genotype-4; it has not been subjected to enough research, mostly because its localization is restricted to the Middle East region and Africa [1]. A significant number of infected patients are at high risk of developing liver cirrhosis and hepatocellular carcinoma. In addition, about 80% of patients with hepatocellular carcinoma (HCC) have underlying hepatitis B or C cirrhosis, and only 10% to 15% are potentially resectable, and the rest are unresectable because of late diagnosis. Moreover, chronic hepatitis C (CHC) does not affect the liver uniformly even with adequate sized biopsies, and cirrhosis may be missed in 15–30% of liver biopsies [2]. This arouses the urgent need to find new biomarkers for diagnosis, treatment, and prognosis of HCV.

During the past decade, much of the accumulated evidence supported the role of interleukins and growth factors in linking inflammation, fibrosis, and tumorigenesis [3]. MicroRNAs (miRNAs) are small noncoding single-stranded RNAs that are 19–24 nucleotides long. They are able to control gene expression at translational or posttranscriptional level, either through degradation or through blocking messenger RNA (mRNA) translation by binding to their 3′-untranslated regions (UTRs). There is strong evidence that approximately 20–80% of transcribed human genes are regulated by miRNAs [4]. miRNAs are present not only in tissue cells but also in body fluids, as a result of passive leakage due to cell death, or they are actively secreted through exosomes from cells [4, 5].

miRNAs are important players in pathogenesis of HCV infection directly by controlling signaling pathways; they play a role in innate and adaptive immune response [1, 2]. miR-122 is the most abundant miRNA in normal liver parenchyma, accounting for more than 70% of the total miRNA in hepatocytes [6]. The binding of miR-122 to the 5′-UTR of HCV genomic RNA is critical for viral replication; it stimulates viral protein translation and protects the uncapped HCV RNA from degradation [7, 8]. Also, upregulation of miR-21 provides feedback inhibition of type I interferon- (IFN-) mediated antiviral response, thereby promoting viral replication [9]. Moreover, miR-21 is one of the first detected oncogenic miRNAs; it controls cell cycle and tumorigenesis [9, 10].

The aim of the present study was first to assess the diagnostic value of plasma miR-122 and miR-21 in Egyptian patients with chronic hepatitis C virus genotype-4 to detect liver fibrosis/cirrhosis development in comparison to other noninvasive indices (APRI) aspartate aminotransferase to platelet ratio index, and aspartate aminotransferase to Alanine Aminotransferase Ratio (AAR). Second aim was to assess their diagnostic value in early stages of hepatocellular carcinoma (HCC) in patients with CLD.

## 2. Material and Methods

### 2.1. Subjects

The research protocol was performed in accordance with the Ethics Committee of the Faculty of Medicine, Alexandria University, Egypt, during the period from April 2014 to April 2016. Informed written consent was obtained from all subjects included in this study.

One hundred and eighty subjects were included in the study; they were divided into 3 groups. Group I included 60 healthy control subjects, group II included 60 chronic hepatitis C (CHC) patients (mostly genotype-4), and group III included 60 patients with hepatocellular carcinoma (HCC) on top of HCV related chronic liver disease (CLD).

All participants were subjected to full medical history, thorough clinical examination, laboratory investigation, abdominal ultrasonography, and ultrasonography guided needle liver biopsy.

### 2.2. Radiological Study

Ultrasound examinations were performed by experienced ultrasonographers. The ultrasound was performed using sonography system (ATL, HDI 3000) with a curvilinear 2.5–5 MHz transducer. Observation and measurement of the liver size, nodularity of the liver surface (which reflects presence of regenerative nodules and fibrous septa indicating cirrhosis), coarseness of the parenchyma, size of lymph nodes around the hepatic artery, patency and flow of veins and arteries, spleen size (which if enlarged can suggest portal hypertension), and hepatocellular carcinoma.

Exclusion criteria included the following: coinfections with hepatitis B virus (HBV) or HIV, organ transplantation, immunosuppression, autoimmune disease, diabetes,* Schistosoma*, and other malignant comorbidities. Also, patients received antiviral therapy, and chemotherapy was excluded.

Diagnosis of CHC was done by assays for HCV antibody testing, by using of a commercial recombinant immunoblot assay (Innolia HCV Ab III; Innogenetics), and confirmed by real-time quantitative HCV RNA PCR (more than 50 IU/ml).

Liver biopsy was taken, with a Menghini needle. The biopsies were then submitted for histopathologic examination. Biopsies were stained with Hematoxylin and Eosin (H and E) and with special stains, namely, reticulin (for reticular fibers), before examination. The stage of fibrosis was evaluated according to Ishak scoring as follows: a score of 0 means no fibrosis, score of 1 is defined as mild to moderate focal areas of fibrosis, a score of 2 was defined as fibrous expansion of most portal areas, with or without short fibrous septa, score of 3 is fibrous expansion of most portal areas with occasional portal-to-portal bridging; score of 4 is fibrous expansion of most portal areas with marked bridging (both portal-to-portal and portal-to-central), and score of 5 is incomplete cirrhosis characterized by marked bridging and occasional nodules and definite cirrhosis [11].

Grading of inflammatory activity within the portal tract and the periportal and lobular regions was also performed as follows: 0 = no histological activity, 1 = minimal lesion, 2 = mild activity, 3 = moderate activity, and 4 = severe activity [11, 12].

All of the HCC cases were diagnosed by abdominal ultrasound and alpha fetoprotein *α*-FP (*α*-FP ≥ 200 *μ*g/mL). Triphasic CT scan examination and Barcelona clinic liver cancers (BCLC) staging and metastatic work-up (CT chest, bone scan) were done for selection of group III patients [12]. The BCLC staging provides beneficial evidence for early biomarker evaluation in terms of diagnostic value and benefit from curative therapies. Patients at a very early stage (Stage 0) and at early stages (Stage A) were selected as optimal candidates for study with single tumors less than 2 cm and Child-Pugh A or single multinodular tumor less than 3 cm in size Child-Pugh A/B, with absence of clinically relevant portal hypertension [11, 12].

### 2.3. Laboratory Investigation

#### 2.3.1. Routine Investigations

Complete blood count (CBC) was determined by automated cell counter (ERMA Inc., Tokyo, Model PCE-210).

Liver profiles—aspartate transaminase (AST) and alanine transaminase (ALT), serum albumin, total bilirubin, plasma prothrombin time (PT), and INR—were measured.

Calculated scores are as follows:

  AAR = AST to ALT ratio.
  APRI (AST to platelet ratio index) = (AST/upper limit of normal)/platelet count (×10^9^/L) × 10.

The Child-Pugh scoring system was performed for all patients, using grades for the following parameters of bilirubin, albumin, prothrombin time, hepatic encephalopathy, and ascites from one to three to a maximum score of 15 [11].

Αlpha-fetoprotein (*α*-FP) was performed by the method described by Chemiluminescence (Immulite 1000, Siemens, Germany).

Lipid profiles—serum total cholesterol (T.C), triacylglycerol (TG), high density lipoprotein cholesterol (HDL-C) and low density lipoprotein cholesterol (LDL-C)—were determined with kits from SENTINEL CH (via principle Eugenio 5-20155 Milan, Italy).

#### 2.3.2. Growth Factors and Inflammatory Biomarkers

Vascular endothelial growth factor (VEGF) was assayed by the method described in the commercial VEGF ELISA kit instructions (Peninsula Inc., USA), while transforming growth factor beta 1 (TGF-*β*1) was assayed according to the method described in commercial kit from Abcam Company (USA). The level of soluble Fas (sFas) was quantitated by the sandwich ELISA kit according to manufacture instructions (Biosource International, Camarillo, California, USA). Absorbance was measured at 450 nm against blank using an ELISA reader (RayBiotech, Canada).

#### 2.3.3. miRNA Quantification by Real-Time qPCR (RT-qPCR)

Blood samples were collected in EDTA tubes, centrifuged at 4000 rpm for 10 min at 4°C, plasma separated, and stored at −70°C.

*RNA Extraction and cDNA Synthesis.* Total RNA was extracted from 200 *μ*l plasma using the miRNeasy extraction kit (Qiagen, Valencia, CA, USA). RNA purity was assessed by the RNA concentration and quantified by NanoDrop Spectrophotometer (NanoDrop ND-1000, United States), followed by analysis using agarose gel electrophoresis. Reverse transcription was performed for cDNA synthesis using the miRNeasy Plasma Reverse Transcription Kit (Qiagen, Valencia, CA, USA) according to the manufacturer's instructions.

*Amplification and Quantification.* The expressions miR-21 and miR-122 were evaluated by RT-qPCR analysis according to the manufacturer's protocol. The housekeeping miRNA SNORD 68 was used as the endogenous control. For real-time PCR, the cDNA template was mixed with SYBER Green Master Mix (Qiagen, Valencia, CA, USA). PCR array plate was enriched with forward and reverse miRNA-specific primers.

The primer sequences were used for miR-122: forward primer: 5′TTGAATTCTAACACCTTCGTGGCTACAGAG-3′ and reverse primer: 5′-TTAGATCTCATTTATCGAGGGAAGGATTG-3′. Primer sequences for miR-21 were as follows: Forward primer: 5′-AGAAATGCCTGGGTTTTTTTGGTT-3′ and reverse prime: 5′-TTGGGAATGCTTTTCAAAGAAGGT-3′.

The housekeeping miRNA SNORD 68 primer sequence is forward: 5′-CTCGCTTCGGCAGCACA-3′ and reverse: 5′-AACGCTTCACGAATTTGCGT3′.

Real-time PCR were performed using an Applied Biosystems 7500 Real-Time PCR System (Foster city, CA, USA) with the following conditions: 95°C for 15 min, followed by 40 cycles at 94°C for 15 s, 55°C for 30 s, and 70°C for 34 s. The cycle threshold (Ct) is defined as the number of cycles required for the fluorescent signal to cross the threshold in real-time PCR.

Expression of miRNAs was reported as ΔCt value. ΔCt was calculated by subtracting the Ct values of miRNA SNORD68 from the Ct values of the target miRNAs. There is an inverse correlation between ΔCt and miRNA expression level, and the lower ΔCt values were associated with increased miRNA. The resultant normalized ΔCt values were used in calculating relative expression values by using 2^−Δ(Ct)^, and these values are directly related to the miRNA expression levels. The 2^−ΔΔ(Ct)^ method was used to determine relative-quantitative levels of individual miRNAs.

### 2.4. Statistical Analysis

The data was collected and entered into the personal computer. Statistical analysis was done using Statistical Package for Social Sciences; SPSS software version 20 for Windows (SPSS Inc., Chicago, IL, USA) was used for data management and data analysis.

Arithmetic mean and standard deviation were used for categorized parameters, and chi square test was used while for numerical data *t*-test was used to compare two groups while for more than two groups ANOVA test was used, followed by post hoc test to determine the level of significance between each two groups, and Duncan method was used, by using the small letters, and the same letters indicate that there was no significant difference, while the different small letters show a significant difference. To find the association between two variables, Spearman correlation coefficient test was used; the level of significant was 0.05.

## 3. Results

**Figure 1:**
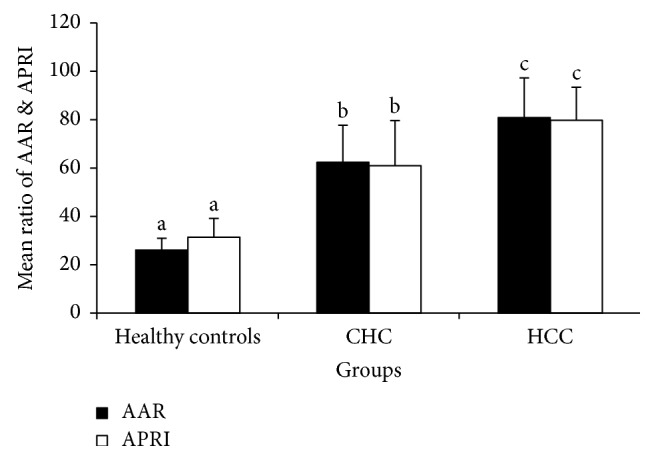
Noninvasive indices AAR and APRI in three studied groups. Values are expressed as means ± SE; mean values within a row not sharing common superscript letters (a, b, and c) were significantly different; *P* < 0.05.

The control cohort consisted of 60 healthy subjects (group I), and there were 60 patients with chronic hepatitis C (CHC) patients (group II); 93.3% of those patients (56 patients) were genotype-4, and only 6.7% were of genotype-1 (4 patients), and 60 patients with CLD were suffering from HCC (group III). The demographic and clinopathologic characteristics of the studied subjects are shown in Table 1 and Figure 1.

According to determined fibrosis score, studied patients were classified into three grades; F2, F3, and F4, and in CHC group 18.3% were F2, 31.7% were F3, and 50.0% were F4, while in HCC group 60.0% were F3 and 40.0% were F4. Staging of HCC patients based on BCLC scoring system revealed that most of the patients were stage A or B, while the minority was stage C. Most of the HCC lesions (67%) were single nodule or two nodules and 59.8% of the lesions were of 3–5 cm size in the biggest diameters (Table 2).

The expression levels of both plasma miR-122 and plasma miR-21 were significantly higher in CHC and HCC in comparison to healthy controls, with more significant increase in HCC compared to CHC group (Table 3).

TGF-*β*1, VEGF, and sFas concentrations were significantly increased in both CHC and HCC groups compared to the healthy controls. As regards TGF-*β*1 values, there was no significant difference between CHC and HCC groups. Moreover, it revealed a significant increase in Child-Pugh class C in CHC patients, while VEGF showed a marked increase in HCC group compared to other groups. However, there was no significant difference of VEGF among the 3 Child-Pugh classes. Also, sFas results revealed no significant difference between CHC and HCC groups. However, there was significant difference in sFas results among the 3 Child-Pugh classes (Tables 4 and 5).

In relation to grade of fibrosis, F4 revealed highly significant increase in TGF-*β*1 in both CHC and HCC groups, and similarly sFas revealed the same increase in same groups. Expression levels of plasma miR-21 and miR-122 were significantly different between the three studied groups, based on the severity of liver cell damage, inflammation, fibrosis, and development of HCC, in which miR-21 showed more significant increase with severity of fibrosis score in CHC and HCC groups, and similarly miR-122 revealed the same result in CHC group while it showed no significant difference between F3 and F4 in HCC group (Table 6).

As regards correlation of miR-122 with other measured variables, there was no correlation with AAR and APRI, and it was positively correlated with other variables especially VEGF. On the other hand, miR-21 was negatively correlated with total cholesterol and positively correlated with other variables especially VEGF (Table 7).

**Figure 2:**
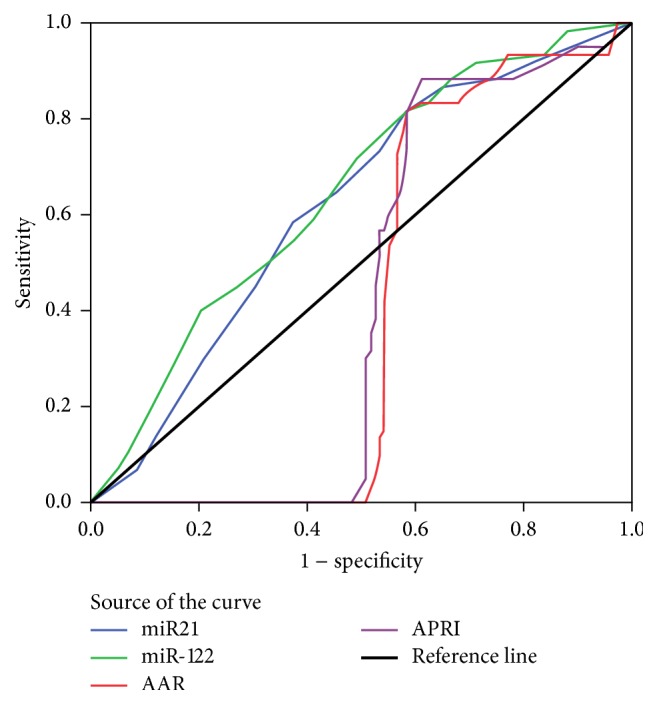
ROC curve was designed for discriminating CHC patients from other groups; results revealed that miR-122 had highest sensitivity, together with miR-21, over other noninvasive indices APRI and AAR.

ROC curve was designed for discriminating CHC patients from other groups, and results revealed that miR-122 had 80% sensitivity, 88% specificity, and 84% accuracy (Figure 2, Table 8).

## 4. Discussion

The incidence of HCC varies widely throughout the world, with rising incidence in Egypt. Most HCC develop in patients suffering from hepatitis infection, which is most prevalent in Middle East region and Africa [1]. In addition, liver pathology develops silently without symptoms, and more than one-third of patients are diagnosed with end-stage liver failure/HCC and die within few months [12]. Therefore, early diagnosis is required for the prevention and treatment of liver cirrhosis and HCC.

The host response triggers an innate immune response against HCV infection, through several mechanisms that include signaling interference, effector modulation, and continuous genetic variation. Those mechanisms are responsible for the persistence of HCV infection [2, 4]. In present study, Genotype-4 was the most common 93.3%. As regards Child-Pugh score; CHC patients results revealed 20.0% of patients were Child A, 21.7% were Child B, and 58.3% were Child C. On the other hand, in HCC patients, 41.7% of patients were Child B and 58.3% were Child C. Most of HCC patients (95%) were stages A and B based on BCLC scoring system.

In this study, the expression level of plasma miR-122 was significantly higher in CHC and HCC groups compared to healthy controls, with more pronounced expression among HCC patients. These findings were in agreement with other studies. They concluded that hepatocytes are primary source of miR-122, and thus it exists copiously in hepatocytes with much lower levels in plasma of healthy individuals. Hepatocyte injury due to HCV infection results in enhanced miR-122 release into the circulation and plasma levels upsurge [6, 13].

In addition, Waidmann et al. concluded that serum miR-122 was significantly reduced in patients with hepatic decompensation as patients with ascites, spontaneous bacterial peritonitis, and hepatorenal syndrome [14]. They elucidated that lower miRNA-122 expressions that occur with more advanced disease are due to higher volume distribution as in patients with ascites. Also, serum miR-122 level might be a marker for hepatic functional capacity in patients with hepatic cirrhosis, whereas, at earlier stages of liver disease, the serum mi RNA-122 level is predominantly an indicator of necroinflammatory activity and hepatic cell injury [14]. Köberle et al. stated that plasma miR-122 expression reveals residual functional liver tissue in patients with end-stage liver disease [15].

Increased expression of plasma miR-122 in patients with HCC was similar to results reported by other studies; Laterza et al. stated that it might be due to its downregulation in HCC tissues and subsequent elevation in circulation of HCC patients [16]. Others reported upregulation of miR-122 expression in HCC; they concluded that patients with high miR-122 had better prognosis and longer survival than others with lower miR-122. They affirmed that miR-122 could modulate p53 activity through the inhibition of cyclin G1 [17, 18].

Moreover, Xu et al. found that miR-122 expression was significantly downregulated in HCC tissues. They suggested that miR-122 acts as a tumor suppressor gene in HCC [19]. Also, Lin et al. in another study stated that cellular mRNA and protein levels of Bcl-w were suppressed by miR-122, which directly targets 3′-UTR, a recognized binding site located in Bcl-w, an antiapoptotic Bcl-2 family member, and subsequently activates caspase-3 with resultant decline in cell viability [20]. Also, they identified Wnt1, as a target related to HCC apoptosis, which was negatively regulated by miR-122 through binding to 3′-UTR of Wnt1 [20]. Tanaka and Arii reported absent expression of miR-122 in serum of HCC would mean lack of differentiation of hepatocytes and consequently poor prognosis [21].

More importantly, Qi et al. found that the levels of miR-122 were significantly reduced in HCC patients postoperatively when compared to the preoperative levels, suggesting that the elevation of circulating miR-122 is likely originated from HCC [22].

There were increased expression levels of miR-122 in CHC group, with more increased expression of miR-122 in stage F3 and F4 fibrosis as compared to F2. On the contrary, Wang et al. reported negative correlation between miR-122 and fibrosis score in hepatitis C patients and also absence of association between miR-122 and ALT serum levels in advanced cirrhosis; they stated that miR-122 did not reflect the liver inflammation activity in CHC [6]. Also, results in present study revealed a significant relationship between fibrosis stages and AAR/APRI, while there was no correlation between miR-122 and either AAR or APRI. Trebicka et al. reported increased miR-122 which was positively correlated with serum transaminases during early stages of fibrosis but was negatively correlated in late stages; they concluded that loss of hepatocytes and progression of fibrosis resulted in drop of serum miR-122 levels [23], while Bihrer et al. found that serum miR-122 levels were comparable between CHC patients with normal ALT levels and healthy controls [24].

In the current study, we reported that plasma miR-21 expression was significantly higher in CHC and HCC groups compared to healthy controls, with more significant increase in HCC group. Increased plasma miR-21 in CHC was in agreement with other studies [10, 25]. They inferred that increased expression in CHC group denoted suppression of HCV-induced production of tumor necrosis factor-*α* (TNF-*α*), interleukin IL-6, and IL-8. As miR-21 decreased HCV induced NF-kB p65 phosphorylation, indicating that NF-kB signaling is also negatively regulated by miR-21 [10, 25].

miR-21 results in the current study revealed positive correlation with serum transaminases levels (ALT and AST), AAR, and APRI in both CHC and HCC groups. Additionally, miR-21 was positively correlated with fibrosis stage. This is in agreement with other studies; they observed miR-21 was positively correlated with fibrotic stage. Similarly, Bihrer et al. found that miR-21 serum levels were strongly correlated with ALT and AST activities, and they inferred that circulating miR-21 level was related to necroinflammatory activity in the liver in patients with CHC [26]. Also, Wang et al. stated release of miR-21 from several different cell types may contribute to the elevation of the serum miR-21 level in patients with CHC, and majority of miRNAs in circulation, including miR-21, are complexed to proteins such as Ago2, implying high stability of miRNAs in plasma [27]. Additionally, Dooley et al. illustrated a relationship between miR-21 and hepatic fibrosis through transforming growth factor *β* 1 (TGF-*β*1), a critical mediator of hepatic fibrogenesis, and it stimulates the expression of miR-21, and that miR-21 reduces the expression of SMAD7, a negative regulator of TGF-*β* signaling [28].

Karakatsanis et al. showed that high levels of miR-21, miR-31, miR-122, and miR-221 expression were correlated with degree of cirrhosis but only high levels of miR-21 and miR-221 were associated with tumor stage as well as lower survival rate than those with low expression levels of miR-21 and miR-221 [29]. Moreover, Xu et al. reported that elevated circulating miR-21 could be also derived from tissue injury in CHC, since patients with CHC might have more serious hepatocytes damage than patients with HCC [30].

Furthermore, increased expression of miR-21 in HCC group would be due to the fact that miR-21 functions as an oncogene and its upregulation promotes malignant cell proliferation and invasion and contributes to evasion of the host immune system [29], and most HCC patients in this study were BCLC stages A and B. Likewise, El Gedawy et al. reported that elevated plasma miR-21 stimulated cell migration and invasion by targeting PTEN (phosphatase and tensin enzyme; part of a chemical pathway that inhibits cell division and triggers apoptosis) and PDCD4 (programmed cell death 4; a tumor suppressor gene) and it could also directly target MAP2K3 (mitogen-activated protein kinase 3) [31].

In addition, in CHC and HCC patients, results displayed significantly lower serum cholesterol levels and triglycerides compared to healthy controls. Total cholesterol was positively correlated with plasma miR-122 and negatively correlated with miR-21; this was explained as miR-122 is a liver specific miRNA whose functions are mainly targeted to maintain liver homeostasis and cholesterol metabolism. miR-122 normally suppresses AMP activated protein kinase (AMPK), principle regulator of metabolism that promotes ATP-generating pathways, reducing fatty acid oxidation while it promotes fatty acid and cholesterol synthesis inside intact hepatocytes, and therefore decreased miR-122 inside hepatocytes resulted in reduced cholesterol synthesis [32]. Also, Elmén et al. reported that injection of locked-nucleic-acid-modified oligonucleotide (LNA anti-miR-122) caused a dose dependent reduction in total serum cholesterol by 30% without apparent toxicity and associated decrease in both HDL and LDL-cholesterol [33]. Moreover, dysregulated lipid metabolism, including eicosanoid pathways, has an impact on inflammatory, immune response and even cancer development [34].

Assessing the degree of liver fibrosis caused by HCV infection was the main tool to diagnose CHC, through calculating noninvasive indices (AAR/APRI) which are the primary indicators clinically. Results of the present study indicated that evaluation of plasma miRNAs expression can be used to evaluate the degree of liver decompensation in CHC. Therefore, a ROC curve was designed to detect biomarker that had the highest efficiency in detecting patients with CHC.

ROC curves revealed that miR-122 had the highest efficiency in prediction of liver cell damage followed by miR-21; AUROC were 0.735 and 0.71 with sensitivity 80% and 79%, specificity 88% and 85%, and accuracy 84% and 82%, respectively, at cutoff value 1.

In the current study, serum TGF-*β*1 significantly increased in CHC and HCC groups compared to healthy controls, with no significant difference in TGF-*β*1 levels observed between CHC and HCC groups. Additionally, patients with Child-Pugh class C score had significantly higher TGF-*β*1 than in Child-Pugh class A and B scores. Likewise, Baghdady et al. reported that serum TGF-*β*1 was elevated in patients with a high Child-Pugh score; they stated that this elevation was caused by increased production and decreased clearance [34]. Elevated TGF-*β*1 promotes liver fibrosis through the activation of hepatic stellate cells (HSCs) and the inhibition of hepatocyte proliferation and regeneration [34]. Also, Yin et al. demonstrated that miR-122 targets different components in the TGF-*β*1 pathway, and dysregulation of miR-122 expression resulted in increased production of TGF-*β*1, a potent cytokine in cell proliferation, differentiation, and migration [35]. Besides, Valva et al. reported that interruption of TGF-*β*1 signaling might improve liver fibrosis and stimulate liver regeneration [36].

In addition, TGF-*β*1 plays a role in HCC development by promoting angiogenesis. Elevated TGF-*β*1 in this study was associated with increased miR-21 expression in HCC group. It indicated increased cell proliferation and tumorigenesis [37, 38]. In HCC, TGF-*β*1, produced in neoplastic cells, would inhibit proliferation of tumor specific cytotoxic T lymphocytes (CTL) and natural killer (NK) cells and stimulate the growth of neoplastic cells, and also it promotes production of VEGF which triggers endothelial cell proliferation, angiogenesis, and tumor metastasis [38, 39].

Moreover, VEGF was significantly higher in HCC group compared to both CHC and healthy control groups; also, there was no significant difference of VEGF among classes of Child-Pugh in both CHC and HCC groups. Analogous results were obtained by El-Mezayen and Darwish; they found no significant difference in VEGF levels between control group and chronic hepatitis C patients; they explained it by the possibility that the two groups had benign liver tissue with no hypoxia and thus is no need for expression of angiogenic factors [40]. On the other hand, Mukozu et al. demonstrated that VEGF was significantly higher in patients with CHC and HCC patients than controls and concluded that VEGF are produced by hepatocytes and contribute to progressive hepatic fibrosis through induced proliferation of HSCs [39].

Results of serum sFas were found to be significantly increased in both CHC and HCC groups compared to healthy controls; with a more significant increase in HCC group. Also, there was a positive correlation between sFas levels and grade of fibrosis as it was significantly increased in fibrosis grades F3 and F4 in both groups. This elevation could be explained by decreased clearance from the damaged liver cells [41]. Increased sFas production may cause a poor CTL-mediated immune response to the viral antigen by preventing the Fas/Fas ligand interaction [42].

Moreover, Wang et al. determined that FasL is a functional target of miR-21; they stated that 3′-UTR region of the FasL mRNA contains conserved miR-21 binding sites, and mutation of these sites canceled miR-21 mediated downregulation of FasL expression [43].

Significantly elevated sFas level in HCC patients emphasized the fact that tumor cells stimulated sFas production to protect themselves from Fas-mediated apoptosis [42]. Thus, elevated sFas levels reflected the degree of liver cell damage; this increase was positively correlated with serum miR-21 in HCC group, and therefore it could be considered as a predictive marker for development of HCC.

In addition, results of the present study revealed positive correlation between miR-21 and both TGF-*β*1 and VEGF in CHC and HCC groups. This was explained by virus-host interactions, in which HSCs became activated as a result of disturbed immune mechanism, and they generated extracellular matrix proteins in response to liver cell injury, followed by subsequent production of TGF-*β*1 and other inflammatory cytokines [36, 37]. Also, *α*-FP levels were correlated with advanced fibrosis, AAR, and APRI, and these results were consistent with Volk et al. that stated that des-gamma carboxyprothrombin (DCP) was more sensitive than *α*-FP in diagnosis of HCC [44]. However, the false negative results with *α*-FP level may be as high as 40% for patients with early stage HCC [12, 44]. The diagnosis of HCC depends on imaging studies obtained using computed tomography (CT).

Consequently, the present study suggested that plasma miR-21 could indicate the degree of liver cell damage, development of malignancy, and prognosis of HCC. Changes in plasma miR-21 most accurately reflected the degree of fibrosis as it was significantly higher in F4 than in F3. Furthermore, it was significantly higher in Child-Pugh C than in Child-Pugh B. Also, it was positively correlated with degree of malignancy. Besides it was strongly correlated with VEGF and *α*-FP compared to other biomarkers, which would predominantly reflect the process of the formation of tumors, more than other measured biomarkers. Thus, plasma miR-21 could be used as an early diagnostic marker for HCC, or maybe combined with VEGF and *α*-FP for a better and early diagnosis.

## 5. Conclusion

HCV infection results in the modulation of miRNA expression and can promote growth factor signaling and cell proliferation. Based on the ROC curves, miR-122 followed by miR-21 had the highest efficiency in prediction of early fibrosis particularly in HCV genotype-4 patients in comparison to other noninvasive indices. Also, they could be utilized in diagnosis of early HCC in patients suffering from CLD in comparison to other measured biomarkers and imaging studies.

Moreover, these miRNAs have an increased advantage over traditional biomarkers since serum/plasma miRNAs are very stable and last for long time and their determination methods are easy to perform. Also, their detection in serum/plasma can clearly define the progression of the disease.

## Figures and Tables

**Table 1:** Demographic and Clinicopathologic characteristics of the three studied groups.

Parameter	Healthy controls (*n* = 60)	CHC (*n* = 60)	HCC (*n* = 60)	*P*
Age	33.9 ± 8.64^a^	35.1 ± 6.7^a^	52.3 ± 10.7	0.002
Gender m/f (%)	36/24 (60/40)	41/19 (68.3/31.7)	42/18 (70/30)	0.106
Albumin (g/dl)	4.11 ± 0.89^a^	3.61 ± 1.42^a^	2.91 ± 1.11	0.003
INR	1.11 ± 0.027^a^	1.41 ± 0.64^b^	1.22 ± 0.49^a^	0.011
T. bilirubin (mg/dl)	1.1 ± 0.28^a^	2.07 ± 1.33^b^	2.74 ± 1.06^b^	0.003
AST (IU/ml)	20.9 ± 7.89^a^	56.22 ± 11.33^b^	107.58 ± 19.3^c^	0.001
ALT (IU/ml)	26.1 ± 7.9^a^	62.11 ± 20.13^b^	97.65 ± 20.11^c^	0.001
Platelets (10^3^/*μ*l)	215.11 ± 60.13^a^	140.22 ± 42.1^b^	120.11 ± 44.1^c^	0.001
AAR	26.11 ± 4.79^a^	62.7 ± 15.11^b^	81.1 ± 16.33^c^	0.011
APRI	31.2 ± 7.98^a^	61.1 ± 18.7^b^	80.1 ± 13.33^c^	0.021
*α*-Fetoprotein (ng/ml)	3.88 ± 1.71^a^	46.25 ± 28.11^b^	217.33 ± 136^c^	0.022
T.C (mg/dl)	192.11 ± 19.36^a^	168.11 ± 13.2^b^	160.01 ± 21.3^b^	0.001
LDL-C (mg/dl)	119.12 ± 10.21^a^	93.68 ± 7.11^b^	90.65 ± 18.11^b^	0.003
HDL-C (mg/dl)	49.11 ± 4.82	47.33 ± 4.78	46.12 ± 9.25	0.092
TG (mg/dl)	175.33 ± 10.31^a^	115.13 ± 11.6^b^	112.75 ± 22.1^b^	0.001

Values are expressed as means ± SE; mean values within a row not sharing a common superscript letter (a, b, c) were significantly different; *P* < 0.05.

**Table 2:** Distribution of patients in CHC and HCC groups as regards Child-Pugh classification, fibrosis score, and staging of HCC.

	CHC (*n* = 60)		HCC (*n* = 60)	
	Number	%	Number	%
*Child-Pugh classification*				
A	12	20	0	0
B	13	21.7	25	41.7
C	35	58.3	35	58.3
*Fibrosis score*				
F2	11	18.3	0	0
F3	19	31.7	36	60
F4	30	50	24	40
*HCC stage*				
A	—	—	25	41.7
B	—	—	32	53.3
C	—	—	3	5

**Table 3:** Plasma miR-21 and miR-122 in the three studied groups.

	Healthy controls	CHC	HCC	*P*
miR-122	0.709 ± 0.28^a^	4.11 ± 3.02^b^	6.73 ± 2.35^c^	0.0036
miR-21	0.998 ± 0.102^a^	6.23 ± 2.41^b^	8.02 ± 3.16^c^	0.001

Values are expressed as means ± SE; mean values within a row not sharing common superscript letters (a, b, c) were significantly different; *P* < 0.05.

**Table 4:** Serum TGF-*β*1 and VEGF and sFas in the three studied groups.

Parameter	Healthy controls	CHC	HCC	*P*
TGF-*β*1 (pg/ml)	28.11 ± 6.91^a^	281.2 ± 158.47^b^	279.2 ± 129.52^b^	0.0001
VEGF (pg/ml)	34.22 ± 17.41^a^	44.1 ± 20.11^b^	216.17 ± 106.29^c^	0.001
sFas (pg/ml)	92.98 ± 16.46^a^	436.22 ± 291^b^	781.46 ± 264.0^c^	0.001

Values are expressed as means ± SE; mean values within a row not sharing common superscript letters (a, b, c) were significantly different; *P* < 0.05.

**Table 5:** Relation between Child-Pugh classification and evaluated biomarkers in CHC and HCC groups.

	Child A	Child B	Child C	*P*
*TGF-β1 (pg/ml)*				
CHC	82.6 ± 42.3^a^	211.3 ± 95.6^b^	302.6 ± 106.6^c^	0.001
HCC	—	228.1 ± 106.3^a^	344.3 ± 81.7^b^	0.005
*VEGF (pg/ml) *				
CHC	38.2 ± 11.6	45.6 ± 21.3	42.6 ± 17.5	0.211
HCC	—	166.2 ± 86.4^a^	206.6 ± 113.6^b^	0.016
*sFas (pg/ml)*				
CHC	298.6 ± 103.6^a^	352.6 ± 98.6^a^	524.6 ± 122.3^b^	0.001
HCC	—	607.5 ± 188.3^a^	840.1 ± 200.1^b^	0.001
*miR-122*				
CHC	2.66 ± 1.62^a^	3.0 ± 1.10^b^	5.1 ± 1.23^c^	0.028
HCC	—	5.8 ± 2.16	7.1 ± 2.58	0.042
*miR-21*				
CHC	4.9 ± 2.03	5.9 ± 2.08	6.8 ± 2.38	0.069
HCC	—	6.02 ± 2.58^a^	9.3 ± 2.57^b^	0.029

Values are expressed as means ± SE; mean values within a row not sharing common superscript letters (a, b, c) were significantly different; *P* < 0.05.

**Table 6:** Relation between grade of fibrosis and evaluated biomarkers in CHC and HCC groups.

	F2	F3	F4	*P*
*TGF-β1 (pg/ml)*				
CHC	102.3 ± 62.3^a^	222.1 ± 69.8^b^	308.3 ± 91.3^c^	0.012
HCC	—	201.3 ± 89.2^a^	299.1 ± 81.6^b^	0.011
*VEGF (pg/ml) *				
CHC	40.2 ± 16.2	45.2 ± 10.8	41.1 ± 8.9	0.451
HCC	—	182.5 ± 69.8	215.9 ± 81.5	0.097
*sFas (pg/ml)*				
CHC	302.5 ± 106.1^a^	385.2 ± 98.5^b^	490.2 ± 106.2^b^	0.012
HCC	—	502.3 ± 108.2^a^	711.3 ± 102.1^b^	0.029
*miR-122*				
CHC	2.3 ± 1.41^a^	3.0 ± 1.71^a^	5.5 ± 1.36^b^	0.039
HCC	—	5.62 ± 2.03	6.8 ± 2.33	0.091
*miR-21*				
CHC	3.8 ± 1.88^a^	4.6 ± 2.53^a^	7.9 ± 2.00^b^	0.027
HCC	—	6.15 ± 2.88^a^	9.8 ± 3.11^b^	0.034

Values are expressed as means ± SE; mean values within a row not sharing common superscript letters (a, b, c) were significantly different; *P* < 0.05.

**Table 7:** Correlation between miR-21 and miR-122 and other variables in both CHC and HCC groups.

	miR-21		miR-122	
	*r*	*P*	*R*	*P*
TGF-*β*1	0.369	0.021^*∗*^	0.325	0.032^*∗*^
VEGF	0.441	0.003^*∗*^	0.406	0.007^*∗*^
sFas	0.391	0.019^*∗*^	0.322	0.039^*∗*^
AAR	0.402	0.012^*∗*^	0.103	0.214
APRI	0.335	0.0289^*∗*^	0.141	0.141
T.C	−0.336	0.0315^*∗*^	0.3550	0.0254^*∗*^

*∗* = significant difference.

**Table 8:** Area under curve and cut off value, sensitivity, specificity and accuracy of miR-21, miR-122, AAR and APRI discriminating CHC patients from other groups.

Marker	Area under the curve	Cut off value	Sensitivity	Specificity	Accuracy
miR-21	0.71	1.00	79.0	85.0	82.0
miR-122	0.735	1.00	80.0	88.0	84.0
AAR	0.559	45.0	68.5	70.1	69.0
APRI	0.550	51.0	65.0	60.0	64.0
